# Water-content related alterations in macro and micro scale tendon biomechanics

**DOI:** 10.1038/s41598-019-44306-z

**Published:** 2019-05-27

**Authors:** Pamela F. Lozano, Mario Scholze, Carsten Babian, Holger Scheidt, Franziska Vielmuth, Jens Waschke, Benjamin Ondruschka, Niels Hammer

**Affiliations:** 1 Department of Anatomy, University of Otago, Dunedin, New Zealand; 2 Institute of Materials Science and Engineering, Chemnitz University of Technology, Chemnitz, Germany; 3 Institute of Legal Medicine, University of Leipzig, Leipzig, Germany; 4 Institute for Medical Physics and Biophysics, Leipzig University, Leipzig, Germany; 5 Vegetative Anatomy, Faculty of Medicine, Institute of Anatomy, Ludwig Maximilian University of Munich, Munich, Germany; 6 Department of Orthopedic and Trauma Surgery, University of Leipzig, Leipzig, Germany; 7 Fraunhofer Institute for Machine Tools and Forming Technology IWU, Dresden, Germany

**Keywords:** Tendons, Biophysics, Ligaments

## Abstract

Though it is known that the water content of biological soft tissues alters mechanical properties, little attempt has been made to adjust the tissue water content prior to biomechanical testing as part of standardization procedures. The objective of this study was to examine the effects of altered water content on the macro and micro scale mechanical tissues properties. Human iliotibial band samples were obtained during autopsies to osmotically adapt their water content. Macro mechanical tensile testing of the samples was conducted with digital image correlation, and micro mechanical tests using atomic force microscopy. Analyses were conducted for elastic moduli, tensile strength, and strain at maximum force, and correlations for water content, anthropometric data, and post-mortem interval. Different mechanical properties exist at different water concentrations. Correlations to anthropometric data are more likely to be found at water concentrations close to the native state. These data underline the need for adapting the water content of soft tissues for macro and micro biomechanical experiments to optimize their validity. The osmotic stress protocol provides a feasible and reliable standardization approach to adjust for water content-related differences induced by age at death, post-mortem interval and tissue processing time with known impact on the stress-strain properties.

## Introduction

Although it is widely present within soft biological tissues forming a major component, water is often overlooked as a factor affecting the biomechanical properties in most testing and standardization protocols. The water content of musculoskeletal tissues appears to be a major driver of stress-strain and failure properties in collagens forming the backbone of ligaments and tendons^1^. This cause-effect relationship, however, has to date only been studied to a minimal extent, and in particular understudied for human tissues, though changes may have vast impact on the validity of the results from mechanical experiments. Typical examples are if human tissues with a different post-mortem interval (PMI) are deployed or if the handling of the tissues causes partial drying of the tissues during storage or even testing. There is still a lack of understanding on the role and influence of water content in the mechanical properties of ligaments and tendons, especially since these can vary depending on external factors such as sex, age, body height and weight^2^ and the anatomical site of the tissue. Approximately two-thirds of a ligament’s composition is water bound in both the formed and unformed matrix^3^, and this may affect drastically tissue mechanics^1,4^. In tendons, it has been shown that a decrease in water content will shorten collagen fibrils within the tendon, which will increase the tensile stresses as a consequence^1^.

The water content of soft tissues, however, can be modified. Techniques such as the osmotic pressure adjustment^5^ have been rendered usefully for macro and nano mechanical experiments. Previous work by our group used the osmotic stress technique, in which the iliotibial tissues had their water content adjusted to the original (native) water content tissue by submerging the sample in polyethylene-glycol solutions before mechanical testing for given times^2,6,7^. The human iliotibial band seems an ideal model for biomechanical experiments assessing the influence of factors such as water content or chemical alteration. It is composed of dense fibrous connective tissue, mostly formed by parallel collagen type I fibers and proteoglycans and is easy to be obtained during forensic autopsies^8,9^.

**Figure 1:**
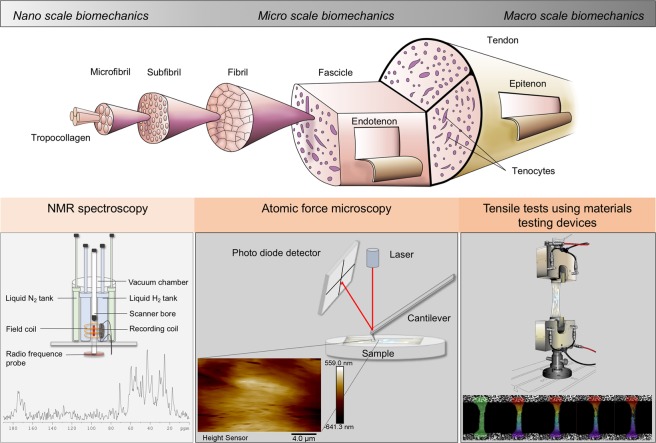
Structural-hierarchical levels of collagen species and a selection of methods to assess tissue biomechanics. Image on collagens adapted from^50^.

Likewise, the osmotic stress technique can be used as an experimental model to alter the tissue water content, thereby quantifying the effects onto mechanical properties. This technique is used frequently for water adjustment for nanoscale biomechanics assessed by NMR spectroscopy^5,10^, and increasingly in macro scale biomechanics^2,6,7^. It may also be relevant for assessing the mechanical properties covering the scale of micro mechanics, represented by atomic force microscopy (AFM) investigations. AFM has a rapidly-growing importance in biological sciences due to its ability to characterize tissues both structurally and mechanically^11–18^. Again, the effects of water content on tissue mechanics derived from AFM appears to be largely understudied and water content related alterations may have implications for the validity of the results in post-mortem tissues or extracellular matrices. Figure 1 summarizes the various hierarchical levels of collagen-rich tissues and a selection of methods used to assess tissue biomechanics.

This given study aimed at quantifying the effect of altered water content on tissue biomechanics at different scales, utilizing the human iliotibial band as a model for collagen-rich tissues and applying the osmotic stress technique. The study aimed at determining if an adaptation of soft tissue water content may form a necessary step for reliable biomechanical testing, and if this applies to both a macro mechanical and a micro mechanical scale.

It was hypothesized that, firstly, altered water content causes altered mechanical properties, implicating that soft tissues should be adjusted before testing (H1). A second hypothesis investigated that at an adjustment of the water content close to the native condition, correlations between anthropological data such as age, PMI, body height and weight become more evident (H2) than would be the case at less physiologic concentrations.

## Materials and Methods

### Sample acquisition

**Figure 2:**
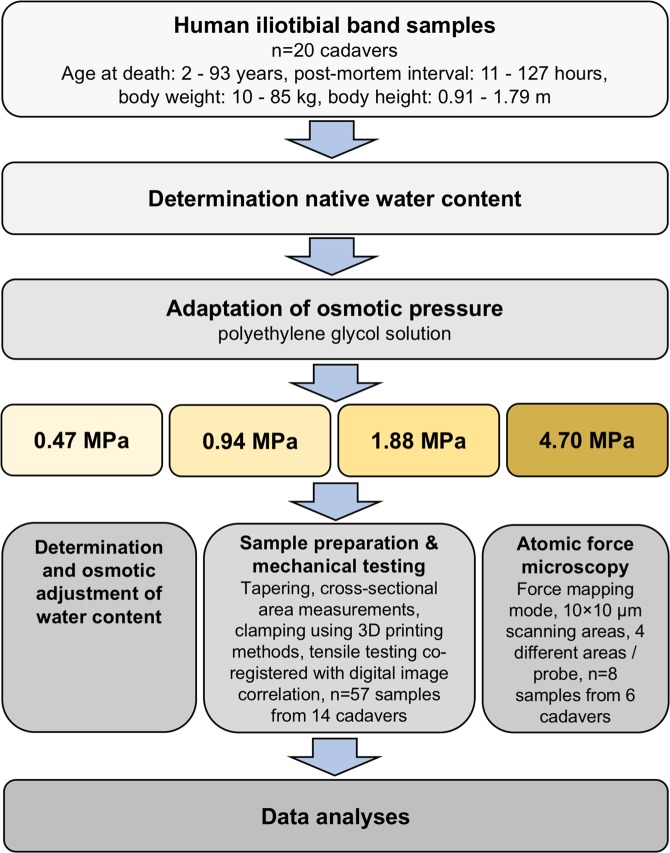
Diagram overview of methods for the iliotibial band samples mechanical testing after osmotic pressure adaptation.

**Figure 3:**
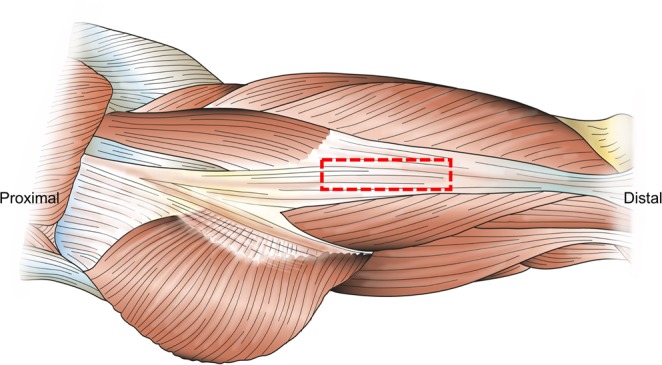
Site of removal of the iliotibial band samples for both macro mechanical and micro mechanical testing.

Iliotibial band samples were obtained from twenty human cadavers (10 females, 10 males) at the Department of Legal Medicine, University of Leipzig, Germany during forensic autopsies after post-mortem intervals between 11 and 127 hours. The Ethics Committee of the University of Leipzig approved the given study (protocol number 156-10-12072010). Informed consent was obtained from the donors’ next of kin in line with the approval of the ethics committee and in accordance to the Declaration of Helsinki. The cadavers’ mean age at death was 56.9 ± 29.7 years (age range 2–93 years), mean height at death 1.61 meters, mean weight at death 62.6 kg and mean body mass index of 22.8 kg/m^2^. The samples were handled as shown previously in Hammer *et al*.^6^ and the experimental steps are summarized in Fig. 2. Fourteen samples (5 females, 9 males) were used for the macro mechanical tests, and six samples (5 females, 1 male) for the micro mechanical experiments. The site and orientation of the samples were kept standardized between all cadavers as shown in Fig. 3 to rule out effects introduced by tissue heterogeneity.

### Water content and osmotic stress protocol

**Figure 4:**
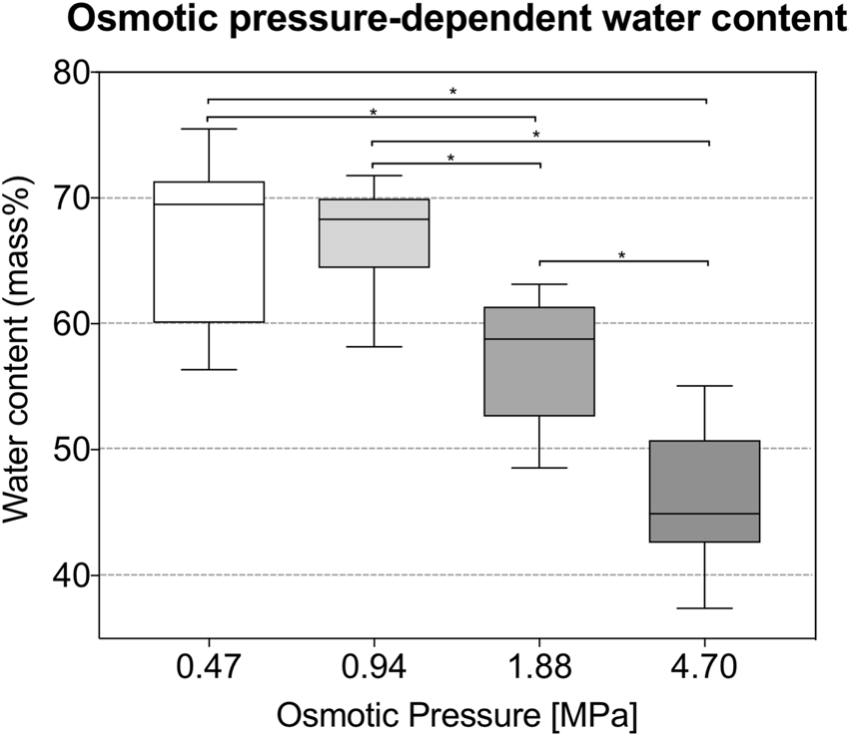
Box plot showing the water content data resulting from osmotic adaption of the human iliotibial band in macro mechanical experiments. Asterisks indicate significantly different (*p* ≤ 0.05) values between osmotic pressures. The boxes indicate the 25-, 50- and 75-percentile, the whiskers the minima and maxima.

Each sample was cleaned carefully to remove any unwanted fat and connective tissue. The water content of the native tissues was obtained by weighing subsamples (*n* = 14, 14 cadavers control group) before and after vacuum-drying for 72 hours. Using an osmotic stress protocol proposed by Schleifenbaum *et al*.^7^, prior to sample preparation and mechanical testing, the subsamples (*n* = 57, 13 cadavers) were placed in 64-mm dialysis membranes (Spectra/Por^®^, Spectrum Laboratories Inc, California, United States; molecular weight cut off 6,000–8,000 Da) and submerged in 20 mM tris-buffered (TBS) polyethylene glycol (PEG) solutions for 24 hours, generating osmotic pressures of 25%, 50%, 100%, and 250% of the osmotic pressure the native tissues are usually exerted to, corresponding to PEG concentrations of 1.25% (0.47 MPa), 2.5% (0.94 MPa), 5.0% (1.88 MPa), and 12.5% (4.70 MPa), respectively (Fig. 4). Small subsamples were obtained from the modified samples and were weighed before $({W}_{wet})$ and after $({W}_{dry})$ vacuum-drying for 72 hours to obtain the water content. For both steps, the water content was calculated using the following formula:

1 $$\phi =\frac{{W}_{wet}-{W}_{dry}}{{W}_{wet}}$$

### Sample preparation and mechanical testing

After being submerged for 24 hours in the TBS-PEG solutions at the four given concentrations, each sample was tapered into a dog-bone shape using a 3D-printed template, which gave a parallel testing length of approximately 8 mm. Each end of the sample was clamped with customized 3D printed clamps. The cross-sectional area of each sample was measured using vinyl polysiloxane (Exahiflex, GC Australia, Sydney, Australia), acting as a cast for the testing area. Each cast was scanned at 1,200 dpi, and the cross-sectional area was obtained with the measuring software (DatInf GmbH, Tübingen, Germany). Prior to placing the sample in the uniaxial testing machine, a stochastic pattern was speckled onto the tissues with standard charcoal pens (Natural Charcoal, Faber-Castell, Stein, Germany).

A Z020 uniaxial testing machine (Zwick GmbH & Co. KG, Ulm, Germany) equipped with a 2.5-kN load cell (Xforce P) along with testControl II measurement electronics was used to test the stress-deformation behavior of the 57 samples from 14 cadavers (5 females, 9 males). Using a crosshead displacement rate of 20 mm/min, and a sampling rate of 100 Hz for force-displacement recording, each sample was preconditioned for 20 cycles in a force range between 2 N and 20 N. Following this, the samples were again unloaded to an initial load of 2 N and in a final cycle stretched until failure. A synchronized digital image correlation system Limess Q400 (LIMESS Messtechnik & Software GmbH, Krefeld, Germany) and ISTRA4D software (Dantec Dynamics A/S, Skovlunde, Denmark) was used to record the optical deformation data during testing.

### Atomic force microscopy

Further, eight iliotibial band samples 1 cm^2^ in size were retrieved (6 cadavers; 5 females, 1 male) of the central part of the samples, washed with PBS after removing the loose connective tissues and embedded in tissue freezing medium. Samples were cryo-sectioned using a Cryostar NX70 Cryostat (Thermo Fisher Scientific, Waltham, Massachusetts, USA) and 8-µm thin sections were incubated with the PEG concentrations described above.

AFM measurements were conducted following 24 h of PEG incubation using a NanoWizard® 3 AFM (JPK-Instruments, Berlin, Germany) with an inverted optical microscope (Carl Zeiss, Jena, Germany). Iliotibial band sections were mounted on microscopy slides. Pyramidal-shaped F tips of Si_3_N_4_ cantilevers were applied (Bio-MLCT, Bruker, Mannheim, Germany) with a nominal spring constant of 0.6 N/m. The spring constant *k* was determined using the thermal noise method^19^. For topographical imaging, AFM contact mode was used. 50 × 50 µm images were acquired using a setpoint of 1 nN and a scanning frequency of 0.3 Hz. For the mechanical measurements, the AFM was operated in a force mapping mode using a set point of 1 nN, z-length of 1.5 µm and an extending/retracting velocity of 10 µm/s for mechanical measurements. 10 × 10-µm wide scanning areas (4 different areas/probe) were chosen from the microscopy image. The resolution was set to 10 × 10 pixels. For each pixel, an individual force-distance curve was acquired. The JPK analysis software was applied to calculate the elastic modulus (here referred as to Young’s modulus of elasticity), using the Bilodeau formula for pyramidal indenter of the Hertz model^20,21^.

### Data analyses

Data processing and statistical comparisons were carried out using MATLAB R2017b (Mathworks, Natick, MA, USA), Microsoft Excel version 16.12 (Redmond, WA, USA) and Prism version 7 (GraphPad Software, Inc., La Jolla, CA, USA). A MATLAB routine was used to process the machine readings and digital image correlation data from macroscopic mechanical testing. Maximum force was evaluated from the machine force-displacement curves and linear stiffness calculated from the initial linear part of these curves by regression analysis. Furthermore, the DIC-data was used to process synchronized load-deformation curves. A nominal strain (virtual point to point line strain) was evaluated in the parallel measurement length of each sample and under inclusion of cross-sectional areas the nominal stress was calculated from synchronized force-readings. The resulting nominal stress-strain curves were further processed for elastic modulus, tensile strength and strain at maximum force. The elastic modulus was calculated by regression analysis in the initial linear part of the stress-strain curve. Tensile strength was defined as maximum of these curves respectively maximum force divided by cross-sectional area. The corresponding elongation at this point was defined as strain at maximum force. Data in the 10% to 90 percentile range were included to minimize the effects caused by data outliers and to achieve comparability between the results. Data were then compared between the four groups with different osmotic pressure using the Kruskal-Wallis test with post-hoc Dunn testing. Specimen data such as age, PMI, body weight and height, cross-sectional area and water content were correlated with the mechanical properties using linear or non-linear correlations. For the AFM data, a one-way ANOVA was used for the normally-distributed data. AFM data were further filtered based on the range of the elastic modulus values in the 10–90 percentile. Here, those single measurements were excluded with elastic moduli variations more than 1,000×, indicating partial volume effects caused by measurements on the underlying glass. *P values* ≤ 0.05 were considered statistically significant.

## Results

### Osmotic stress adaptation led to significantly different water content values compared to the native and the reference condition

The 1.88-MPa (100%) osmotic pressure PEG solution resulted in a water content of 57.2 ± 5.0%, which was the closest to the native water content of 54.4%. Smaller osmotic pressures led to consistently higher water concentrations in the tissues, and larger pressures led to consistently lower water concentrations. The water content values varied significantly between the 0.47 vs. 1.88 MPa (*p* < 0.001), 0.47 vs. 4.70 MPa (*p* < 0.001), 0.94 vs. 1.88 MPa (*p* < 0.001), 0.94 vs. 4.70 MPa (*p* < 0.001), and the 1.88 vs. 4.70 MPa (*p* < 0.001) groups, respectively (see Fig. 4). The cross-sectional areas averaged 4.9 ± 1.3 mm^2^, 4.3 ± 1.2 mm^2^, 4.2 ± 0.8 mm^2^ and 4.1 ± 1.2 mm^2^ for the 0.47-, 0.94-, 1.88- and 4.70-MPa groups, respectively, without significant difference (p = 0.366).

### Macro mechanical properties of human iliotibial band were largely water-dependent; lower water content increased elastic modulus and ultimate tensile strength values

**Figure 5:**
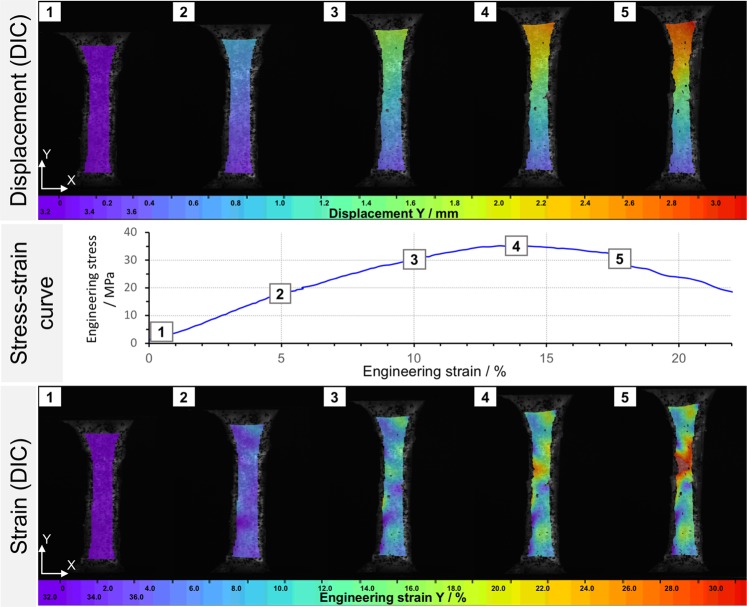
Example of a displacement and strain evaluation by digital image correlation at different steps of the nominal stress-strain curve in a uniaxial tensile test. The corresponding engineering strain curves for data evaluations were calculated by a point-point / line strain in the parallel length of each sample during tensile loading.

**Figure 6:**
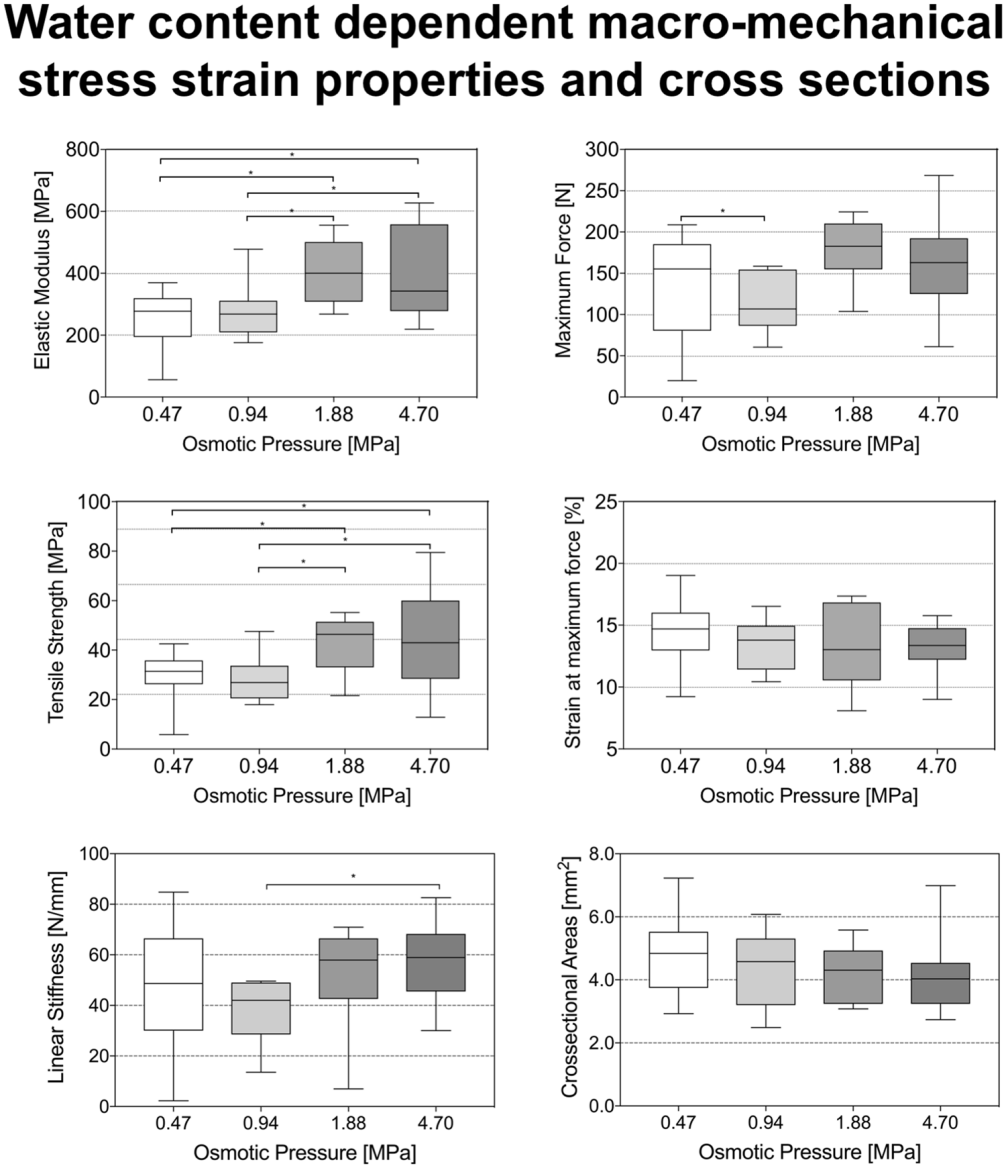
Boxplot presentation of the data obtained from the mechanical testing resulting from osmotic adaptation of the iliotibial band. Asterisks indicate significantly different (*p* ≤ 0.05) values between osmotic pressures. The boxes indicate the 25-, 50- and 75-percentile, the whiskers the minima and maxima.

Elastic modulus, tensile strength, and maximum force showed significantly different mechanical values between the groups with the different water osmotic pressures. Typical image correlation data including displacement and strain are given in Fig. 5. Regarding the mode and site of failure, no differences were observed for the four groups. Figure 6 summarizes the osmotic pressure-dependent stress-strain data. Decreased water content led to higher elastic moduli. The mean elastic modulus was significantly lower at lower osmotic pressures (*p* = 0.011 for 0.47 vs. 1.88 MPa; *p* = 0.005 for 0.47 vs. 4.70 MPa; *p* = 0.034 for 0.94 vs. 1.88 MPa; *p* = 0.020 for 0.94 vs. 4.70 MPa). Similar observations were made for tensile strength. Here, higher osmotic pressures with resulting lower water content of the tissues gave increasing values, respectively.

Comparison of tensile strengths at 0.47 and 0.94 MPa and of 1.88 and 4.70 MPa yielded similar and non-significantly different results. However, tensile strength was significantly lower in the 0.47 vs. 1.88 MPa (*p* = 0.040), the 0.47 vs. 4.70 MPa (*p* = 0.016), the 0.94 vs. 1.88 MPa (*p* = 0.028) and the 0.94 vs. 4.70 MPa (*p* = 0.011) group.

### Correlations exist between elastic modulus and age at water contents close to the native condition only

**Figure 7:**
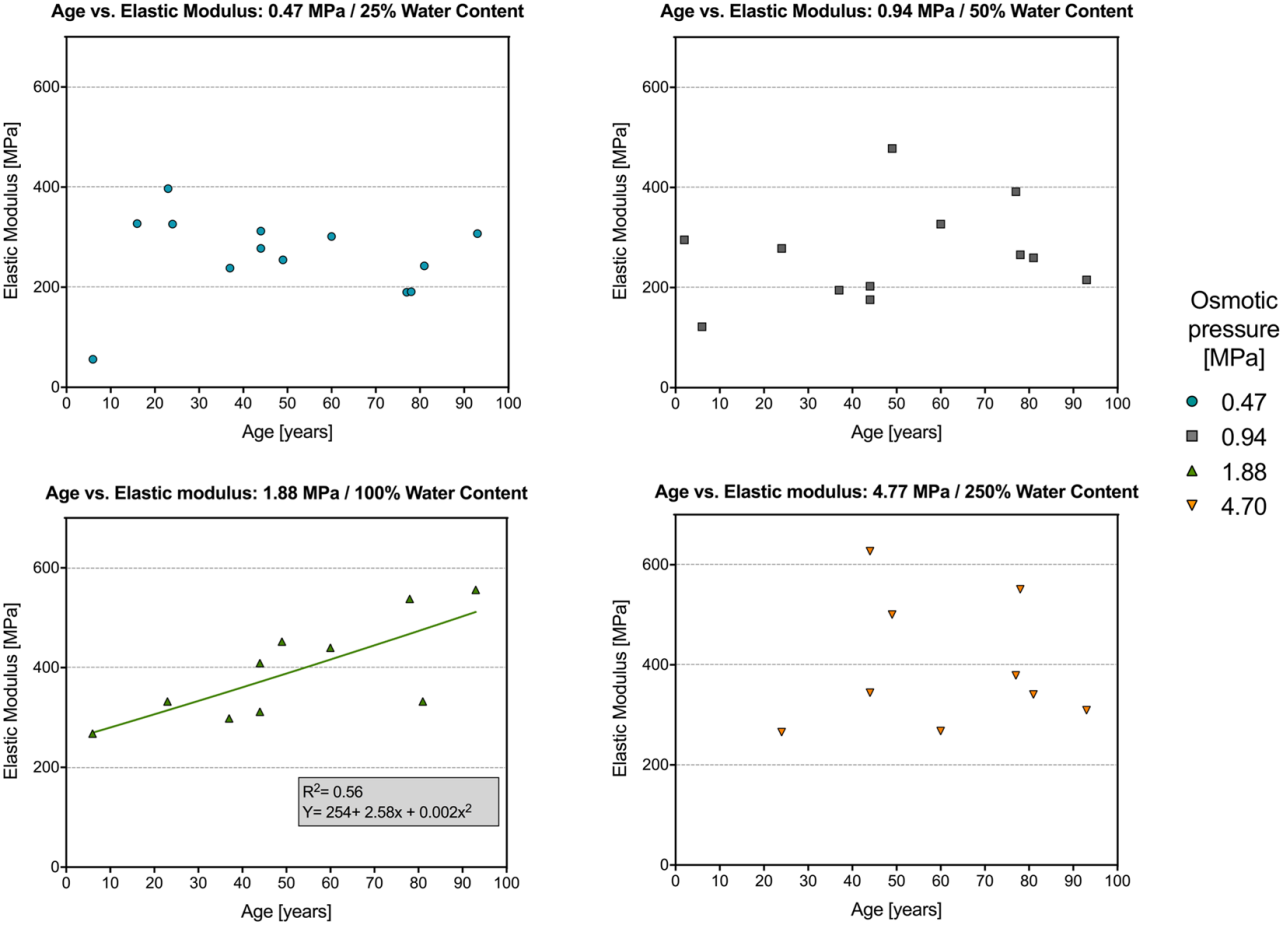
Non-linear representation of age vs. elastic modulus. R^2^ and an equation given for the correlation found in the 1.88-MPa osmotic pressure group.

Of the data found between anthropological data and mechanical properties, an important and significant correlation exists for the 1.88-MPa group between elastic modulus and age with R^2^ = 0.56 (Fig. 7). The best non-linear fit found for this information was given in form of a quadratic formula, in which *y* represents elastic modulus and *x* corresponds to the donor’s age of the tissue:

2 $$y=254+2.58{\rm{x}}+0.002{{\rm{x}}}^{2}$$

Regarding further correlations between further anthropological and death related parameters, the following statistically significant correlations were observed for the 0.47-MPa group: body height vs. elastic modulus (r = 0.54, p = 0.026) and body weight vs. maximum force (r = 0.52, p = 0.031). For the 0.94-MPa group, the following correlations were found: body weight vs. maximum force (r = 0.63, p = 0.014), body height vs. tensile strength (r = 0.67, p = 0.009), body height vs. maximum force (r = 0.65, p = 0.011), PMI vs. elastic modulus (r = 0.64, p = 0.013) and PMI vs. tensile strength (r = 0.51, p = 0.004). No other significant correlations were found for these and the other osmotic pressures. Moreover, in particular the correlations of PMI and mechanical values have shown particularly low values of determination (R^2^), ranging between 0.26 and 0.41. In all other groups, PMI in the range of this study seemed less decisive for any of the mechanical parameters. Related scatter plots are given in Figs S1–S4.

### Micro mechanical properties of the human iliotibial band were largely water-dependent; lower water content increased elastic modulus in atomic force microscopy

**Figure 8:**
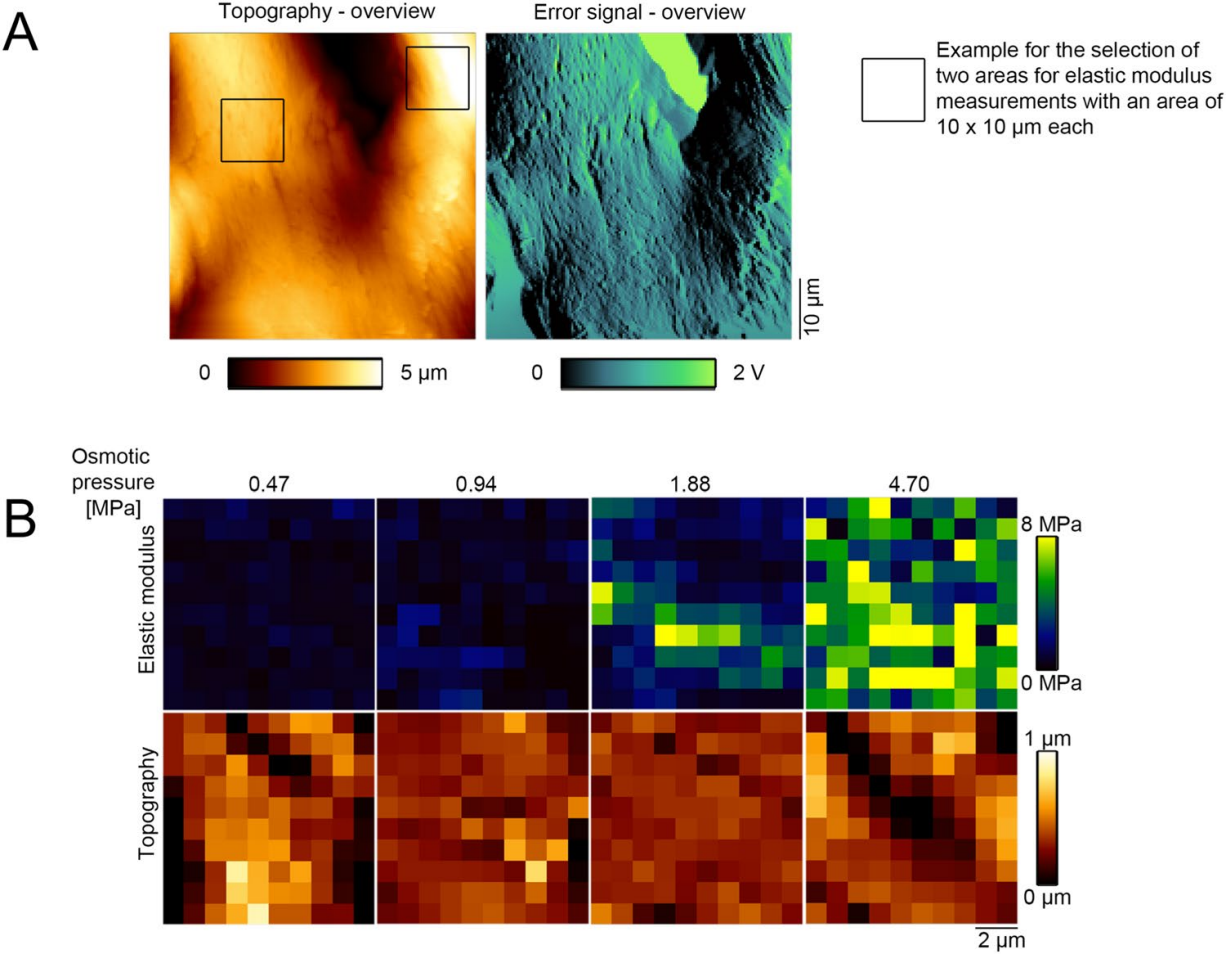
Images from atomic force microscopy of human iliotibial band samples. (**A**) The images show the height and error signal of sample sections in contact mode. (**B**) Pictures are representatives of elasticity maps under the respective osmotic pressure. Elastic modulus does at least in part follow topography of the respective samples.

**Figure 9:**
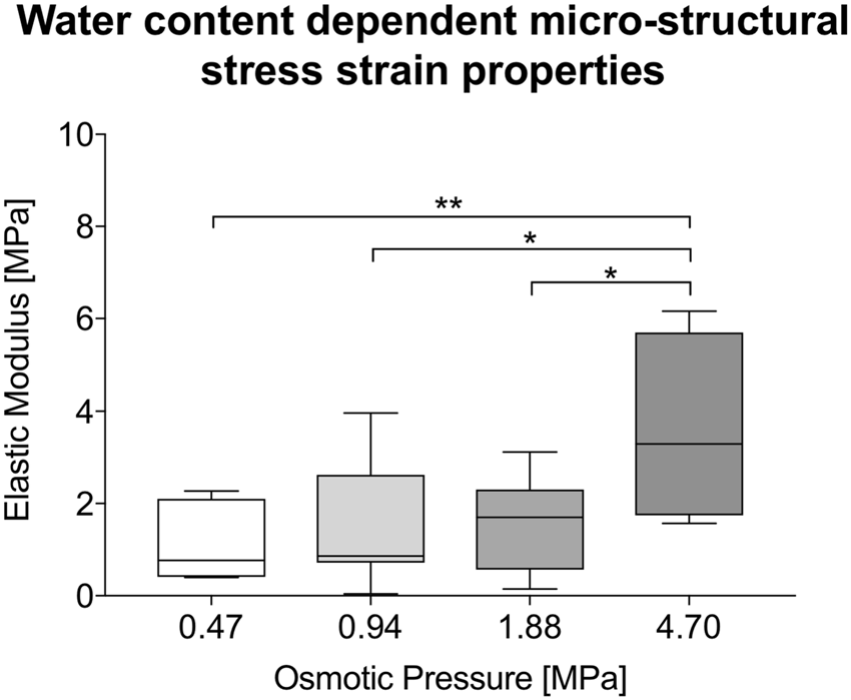
Boxplot representation of the data obtained from micro mechanical testing in AFM following the osmotic adaptation of the human iliotibial band. Asterisks indicate significantly different (*p* ≤ 0.05) values between osmotic pressures. The boxes indicate the 25-, 50- and 75-percentile, the whiskers the minima and maxima.

In line with the macro mechanical tensile experiments, the measurements from AFM confirmed that the water content significantly influences tissue mechanics on a micro structural and -mechanical level. Figure 8 shows topography-based data obtained in contact mode and the resulting mechanical values after osmotic adaptation. The elastic modulus averaged 1.04 ± 0.81, 1.65 ± 1.40, 1.57 ± 1.05 and 3.58 ± 2.08 MPa, for the 0.47, 0.94, 1.88 and 4.70-MPa osmotic stress groups, respectively. The values varied significantly between the 0.47 vs. 4.70 MPa (p = 0.006), the 0.94 vs. 4.70 MPa (p = 0.028) and the 1.88 vs. 4.70 MPa (p = 0.027) osmotic stress groups, respectively (Fig. 9).

## Discussion

Using a highly standardized protocol for biomechanical testing, this study gives experimental evidence that the water content of soft tissues should be adjusted to minimize unpredictable effects on tissue biomechanics and finally to give reliable results close to the living state.

Water content plays a crucial role in the biomechanics of soft tissues^1,6,22^. The purpose of this experiment was to determine if an adaption of tissue water content is necessary prior to biomechanical testing, both on a macro and a micro mechanical scale. By using the osmotic stress technique, the water content of human iliotibial band specimens was altered in a standardized manner to understand its influence on the mechanical properties and to assess if the water content should be standardized for mechanical tests. Following the steps proposed elsewhere^6^, to reduce a spread of the results, all samples were obtained from the cadavers at the same anatomical site were loaded uniaxially, and material slippage during testing was reduced by using 3D-printed clamps, designed by our laboratory^23^.

Mechanical tests of human soft tissues are carried out frequently in a range of hierarchical levels, as exemplified in Fig. 1. A number of AFM studies can be found in recent literature, assessing individual collagen fibrils^11,13,14,24–29^ or composite soft tissues involving a significant collagen backbone^12,16,25,30,31^. Structural changes^25,32,33^ of collagens or collagen remodeling^34^ are assessed on the level of collagen fibrils.

Svensson and coworkers^18^, in their study on human patellar tendon, found that collagen fibril and fascicle mechanics are insensitive to environmental salts at different concentrations when individual collagen fibrils are strained 4% longitudinally along the collagen fiber axis. Our results contrast these previous findings, showing different mechanical properties when tissues are exerted to different osmotically active substances, effectively changing the tissue water content. No study to date has shown this direct impact of different concentrations of osmotically active substances on tissue hydration. Further, it remains unclear if using isotonic saline is effective at restoring and maintain soft tissue water content though this is the most common approach in mechanical testing. Consequently, it is difficult to determine if Svensson *et al*.^18^ have effectively altered the water content in their tendon model following the mounting of the tissues which involved denaturation caused by the chemicals.

Our mechanical data presented here indicate that both cross-section dependent and non-cross-section dependent mechanical properties are altered as a consequence of altered water content. For the cross-section dependent values such as elastic modulus and tensile strength one may well explain increasing values as the consequence of drying-related shrinkage of the tissues, to the end that more collagen fibers are situated in an area with a given similar cross-sectional area. It needs to be emphasized here that with the given dog bone shape templates only two of the three dimensions of the tissues were altered, length and width, whereas the sample thickness was left unaltered. Thickness would consequently decrease in line with the other dimensions and not be compensated for. This finding in itself would already form a rationale for considering and adjusting the water content for standardization procedures. However, it could be shown that also those values which were not cross-section dependent such as maximum force and linear stiffness yielded significantly different values comparing the various osmotic pressures and related water content. These findings may be interpreted that beyond potential alterations in the cross-section, the state of hydration directly influences the load-deformation behavior of collagen-rich tissues. Similar findings have been reported by Safa, *et al*.^35^, who found significant alterations in modulus and equilibrium stress of rat tail tendon fascicles following an 8-hour submersion in 0.9% saline solution. Their study consequently recommended against using saline buffer solutions in favor of PEG-based solutions^35^.

### Water content is a driver for altered mechanics in collagen-rich soft tissue

A comparison between the tissues adapted osmotically revealed that mechanical properties were affected by the amount of water in the tissue, in both macro mechanical testing and AFM. It was observed that lower water contents induced higher elastic moduli, and tensile strength in line with Adeeb, *et al*.^36^ and Safa, *et al*.^35^. Nevertheless, there was no significant change in strain at maximum force at the various water contents investigated in the macro mechanical tests carried out here. The water-dependent change of the mechanical properties can be explained by the high attraction of water molecules to the negatively-charged proteoglycans and the triple-helix collagen fibers found in the matrix of the iliotibial band. Lower water contents will cause less movement of the collagen fibrils and fibers, causing friction and energy loss in the form of heat, resulting in a stiffer tissue and more loss of water^37^. Contributing with the previous, a change in the water content will also influence the thickness of the collagen fibers^35,38,39^, making them more prone to stiffening and changes in their viscoelastic behavior at the hierarchical levels presented here. This functional relationship of the iliotibial band is in line with the tissue acting as a stabilizer for both the hip and knee joint, given effective two-leg propulsion requires a significant amount of energy storage and force regain capacity^40,41^. The interfascicular matrix of this tendon oversees its stretching and recoiling properties. In this space, non-collagenous proteins such as elastin and lubricin are found^42^. These proteins aid in tendon development by controlling fiber and fascicle gliding and promoting elasticity^42^. If water content is not controlled *in situ*, the tendon could be in risk of injury due to an unexpected change in its mechanical properties.

In addition, a decrease in water content in ligaments increases the release of collagenase^43^. This decreases the quantity and density of collagens, as well as weakens the tensile properties of the ligament due to the induced disorganization of the collagen fibers^44^.

The importance of the osmotic stress technique on a macro and micro mechanical level is underpinned since its application helps to avoid changes in mechanical properties of the tissue and can help in mimic the properties of native tissue.

The submersion time of the tissue may also affect the resulting water content. In our experiment, a constant submersion time of 24 hours in TBS-PEG solution was applied according to own experiences. PEG itself as the osmotically-active substance appears to be well suited, as shown macro^2,6,7^ and nano mechanically^10,45^. Zhang, *et al*.^46^, preserved cortical bone in saline solution for 3, 10, 36, and 60 days. The results of this study^45^ showed a loss in tissue elasticity and ultimate strength, likely as a consequence of deteriorated organic matrix in the bone, consisting of collagens, during longer storage. This effect was seen as early as 3 days after submersion, thus, a shorter preservation time in solutions could give more accurate results regarding native tissues without additional errors introduced by the duration of the tissues for the purpose of osmotic adaptation or storage. In another experiment conducted by Gratz, living human fascia lata tissues from a similar site had a preservation time between two and eighteen hours in saline solution^8^. In the biomechanical experiments, tensile strengths of 43 to 54 MPa were obtained^8^, which agrees with the previous native tissues from our study. According to Hammer, *et al*.^47^, native iliotibial tract tissue has an elastic modulus of 369 ± 192 MPa. In this experiment, an osmotic pressure of 1.88 MPa resembled the tissue at the native condition and resulted in an elastic modulus of 398 ± 99 MPa. Thus, in line with our first hypothesis, alterations in water content change the mechanical properties of soft tissues. Consequently, the water content of collagen-rich tissues such as the iliotibial band or other ligaments or tendons should be implemented to modify soft tissues before testing to improve the repeatability of the data and to account for potential shifts in water content post-mortem. To our surprise, changes induced by the PMI up to five days have not induced significant alterations in the here observed mechanics, likely also due to our attempt to standardize water content of the samples.

### Correlations between anthropological data, mechanical properties, and water content exist at water concentrations close to the native condition

Once determined that an osmotic pressure of 1.88 MPa yielded a water content similar to the native tissue, the samples of this group were tested for correlations between anthropological data and mechanical properties. A correlation between age and elastic modulus was found, but not for the other groups of osmotic pressure. With an age range of 2–98 years, it was observed that a higher elastic modulus was obtained for older donors, indicative of a stiffening of the tissues with higher age. A loss of proteoglycans is observed in elder people; thus, water content will also decrease since water molecules bind to proteoglycans, making tendons stiffer^48^. Osakabe, *et al*.^49^ explained that extracellular components such as elastin, fibrillin, and collagen decrease with age. Using lumbar yellow ligaments, a relationship between age and stiffness was observed: the older the person, the stiffer the tissue. In addition to the loss of proteins that provide elasticity, it was found that mineral contents increased with age, promoting stiffness even more. Hammer, *et al*.^47^ agree with the previous by showing that elastic modulus is lower in younger tissues. Iliotibial tracts from donors younger than 44 years old were used in this former experiment. Due to the large variation in age at death in our given experiment, a mean age at death corresponded to 57 years, which is close to the age group investigated in our previous works^47^.

Thus, in support of our second hypothesis, water content influences mechanical properties at a different extent. Other correlations were found for higher water contents and mechanical properties, nevertheless, these were inconclusive.

Correlations for other anthropological characteristics could not be obtained for the 1.88-MPa group due to the limited sample size, possible alteration of the biochemical composition of the tissues due to their PMI, and potentially differences between male and female’s metabolism. Moreover, given only a limited number of samples was available in particular for the AFM experiments, this study could not assess sex-specific difference on altered water content in particular for the micro mechanical properties. If a standardized procedure is applied in future testing, more concrete data could be obtained to understand the influence of water content, anthropological data, and mechanical properties in more detail.

## Conclusions

This study showed that there are water-content related alterations in tendon biomechanics on a macro and micro mechanical level. The results obtained in this study underline the importance of measuring and adjusting the water content of biological tissues, with implications on the accuracy and repeatability of tensile biomechanical testing of collagen-rich soft tissues such as the iliotibial band. Utilizing the osmotic stress technique to adjust the tissues’ water content as an integral part of biomechanical testing is highly encouraged. Correlations between age and elastic modulus were found for water content close to the native condition; nevertheless, more research is needed in order to understand the effect of water content in other mechanical properties.

## Supplementary information

Supplement Figures
