# Supplementary material for: Water-content related alterations in macro and micro scale tendon biomechanics
Source: Sci Rep. 2019 May 27;9:7887. doi: 10.1038/s41598-019-44306-z (PMC6536550; doi:10.1038/s41598-019-44306-z)

**Water-content related alterations in macro and micro scale tendon biomechanics**

Pamela F. Lozano ^1,^*, Mario Scholze ^1,2,^*, Carsten Babian ^3^, Holger Scheidt ^4^, Franziska Vielmuth ^5^, Jens Waschke ^5^, Benjamin Ondruschka ^3^, Niels Hammer ^1,6,7^

1. Department of Anatomy, University of Otago, Dunedin, New Zealand
2. Institute of Materials Science and Engineering, Chemnitz University of Technology, Chemnitz, Germany
3. Institute of Legal Medicine, University of Leipzig, Leipzig, Germany
4. Institute for Medical Physics and Biophysics, Leipzig University, Leipzig, Germany
5. Vegetative Anatomy, Faculty of Medicine, Institute of Anatomy, Ludwig Maximilian University of Munich, Munich, Germany
6. Department of Orthopedic and Trauma Surgery, University of Leipzig, Leipzig, Germany
7. Fraunhofer Institute for Machine Tools and Forming Technology IWU, Dresden, Germany

Word count: 4412, Abstract 200

**Corresponding author**

Niels Hammer, M.D., Department of Anatomy, University of Otago, Lindo Ferguson Building, 270 Great King St, Dunedin 9016, New Zealand; Phone: +64 3 479 7362, Fax: +64 3 479 7254

Email: nlshammer@googlemail.com

* First and second author contributed as equally to the manuscript

**Conflict of interest statement**

The authors declare that no conflict of interest exists related to the given study, including

honoraria, travel to conferences, consultancies, stock ownership (excluding publicly owned

mutual funds), equity interests, and patent-licensing arrangements (particularly if a commercial

product is noted in the article).

**Supplement figures**

**Figure S1:** Age vs. mechanical properties

**
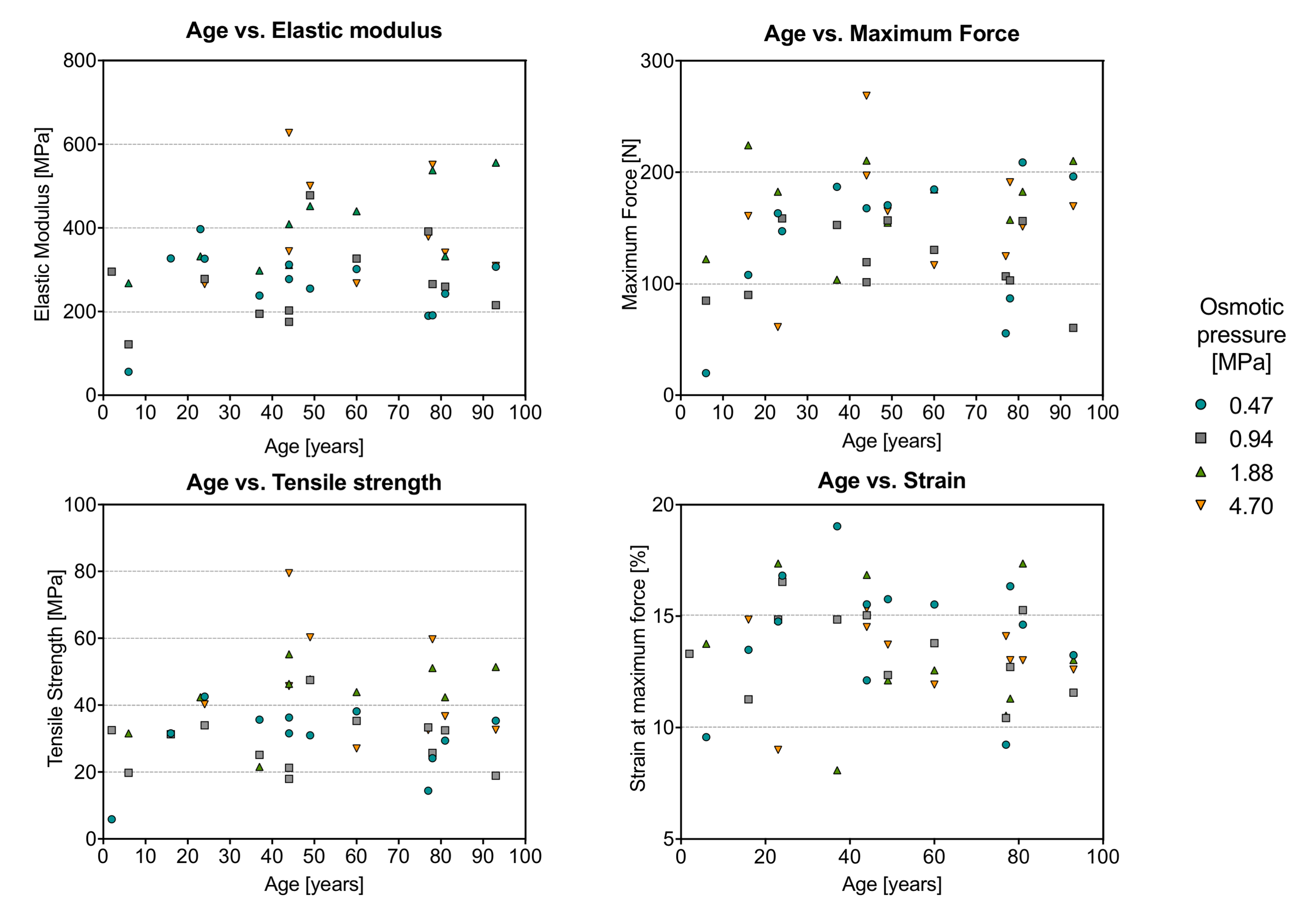
**

**Figure S2:** Body weight vs. mechanical properties

**
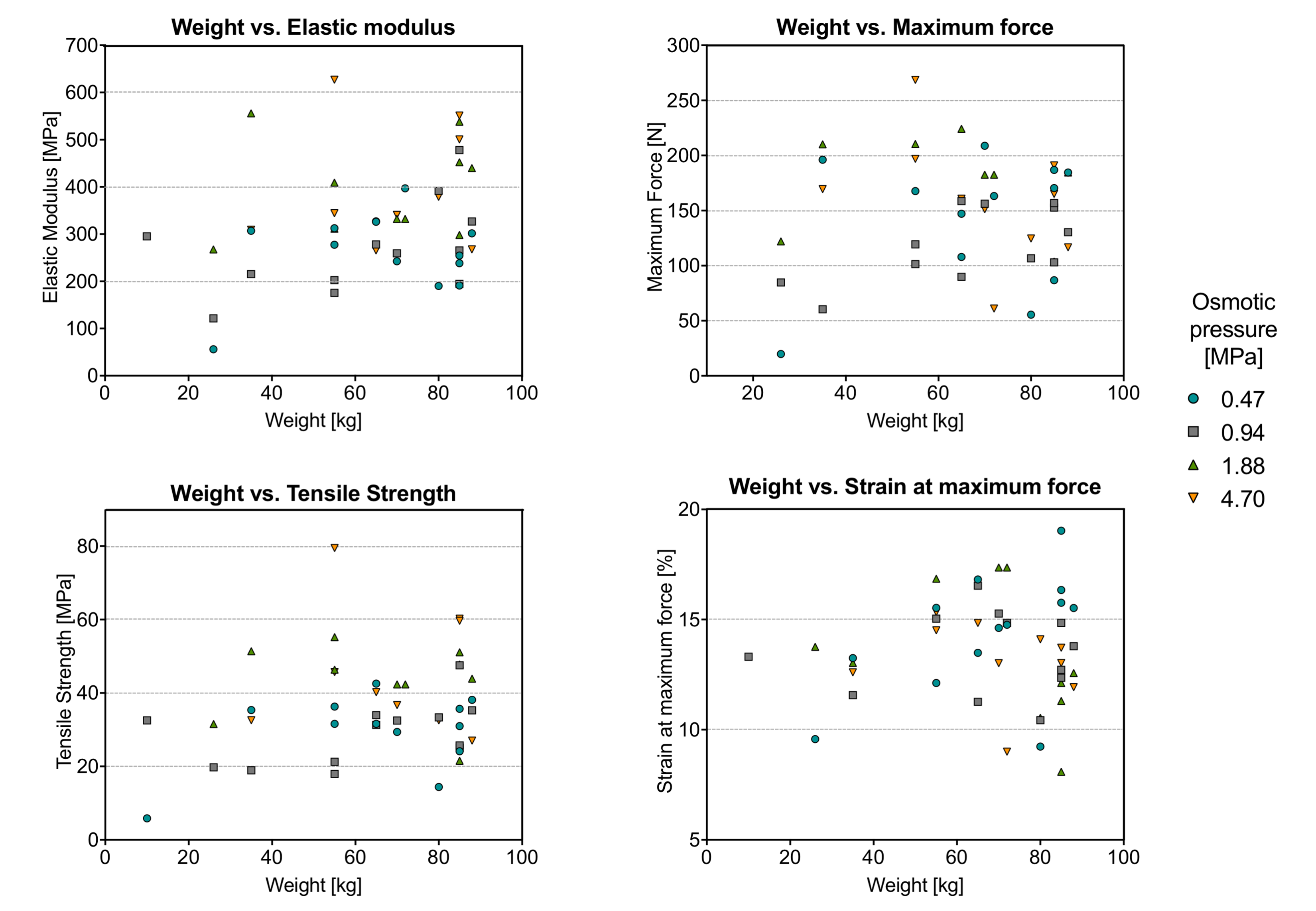
**

**Figure S3:** Body height vs. mechanical properties

**
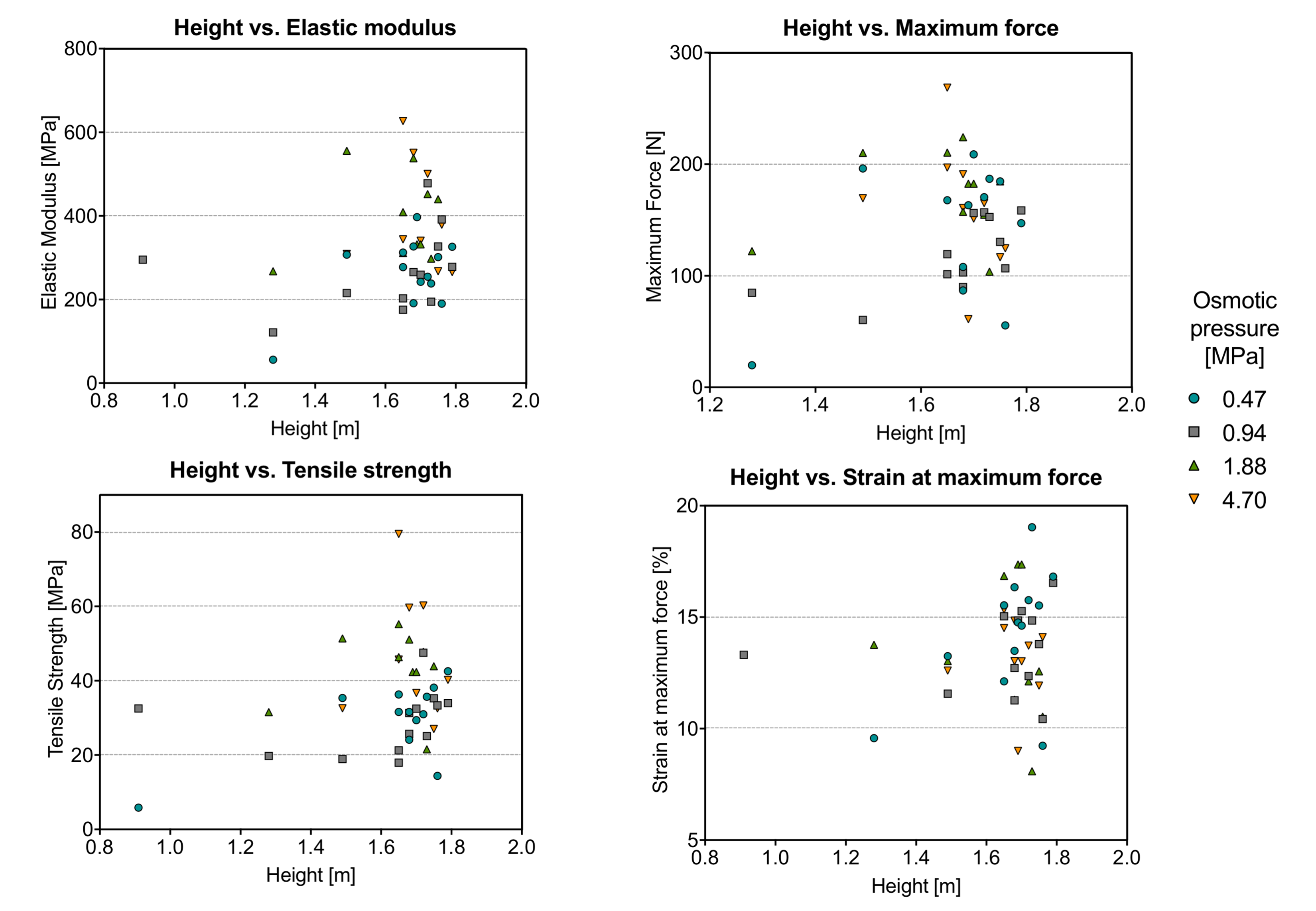
**

**Figure S4:** Post mortem interval vs. mechanical properties


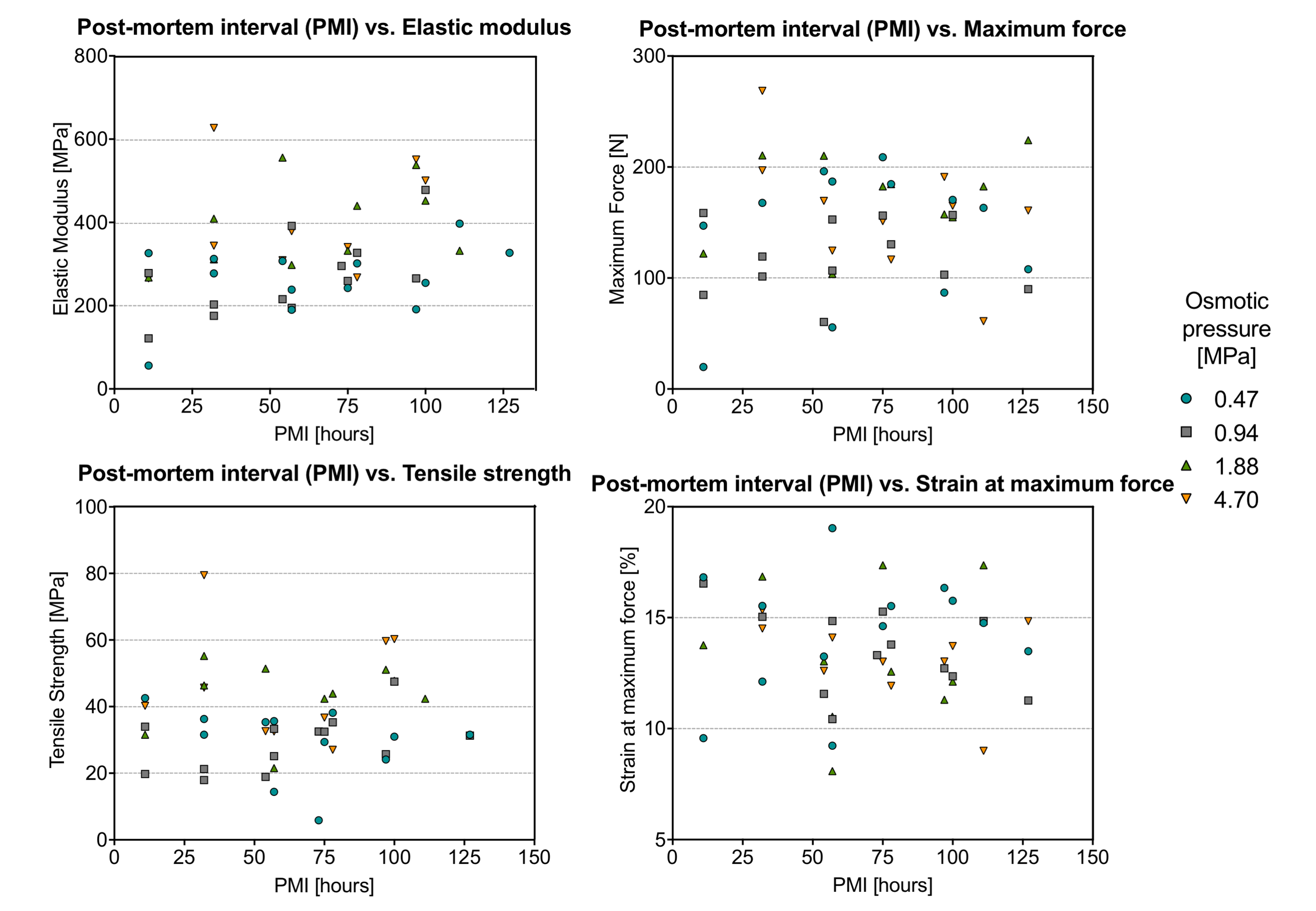

Supplement: Supplementary file 1 — Supplement Figures [file 41598_2019_44306_MOESM1_ESM.docx]
